# The Ablation of Thyroid Nodule’s Afferent Arteries Before Radiofrequency Ablation: Preliminary Data

**DOI:** 10.3389/fendo.2020.565000

**Published:** 2021-02-11

**Authors:** Chiara Offi, Sara Garberoglio, Giovanni Antonelli, Maria Grazia Esposito, Umberto Brancaccio, Claudia Misso, Edoardo D’Ambrosio, Daniela Pace, Stefano Spiezia

**Affiliations:** ^1^Department of Endocrine and Ultrasound-guided Surgery, “Ospedale del Mare”, Naples, Italy; ^2^Division of Endocrinology, Diabetology and Metabolism, Department of Medical Sciences, University of Turin, Turin, Italy; ^3^Department of Endocrinology, Valmontone Hospital, Valmontone, Italy

**Keywords:** radiofrequency ablation, thyroid nodules, technique, ultrasound, thermal ablation

## Abstract

Induced radiofrequency thermal ablation is the cytoreductive treatment of symptomatic benign thyroid nodules, metastatic and recurrent thyroid tumors and papillary thyroid microcarcinomas. It is a safe and effective alternative to surgery and it allows to obtain satisfactory results in terms of volumetric reduction of the nodule with significant improvement in the quality of life. The trans-isthmic approach and the moving shot technique are the two basic techniques; however, an advanced technique, artery-first feeding radiofrequency ablation, has been developed and validated. We have prospectively included 29 consecutive patients who have undergone radiofrequency ablation (Group A) or artery- first vRFA (Group B). All included patients had a diagnosis of benign nodular goiter and they underwent a single session of radiofrequency ablation. All patients followed a follow-up program at 1 month, 3 months, and 6 months. Continuous variables (age, TSH value, basal volume of nodule, used Joule, time in second of the procedure, nodules’ volume at 1-, 3-, and 6- months of follow-up and percentage of volume reduction at 1-, 3-, and 6- months of follow-up) were described as mean, standard deviation and range, while categorical variables (gender, nodule structure and nodule vascularization) were described as number of cases and percentage. Independent samples t-test were performed to compare the continuous variables. A Test of Proportions was applied to the categorical variables. The Fisher’s exact test was used to analyze the gender. Statistical significance was considered in case of p-value <0.05. Solid structure and spongiform structure showed statistic differences with p-values of 0.022 and 0.023 respectively between two groups. The percentage of reduction at 1 month did not show a significant difference between two groups; instead, the percentage of volume reduction was decreased mostly in the Group B at 3 months and 6 months of follow-up with a p-value of 0.003 and 0.013, respectively. The Joules/energy used showed a statistically significant difference (p-value=0.05), more energy must be used in vascular radiofrequency ablation. These data allow us to hypothesize that vRFA may improve the effectiveness of the procedure, allowing for a reduction in volume more quickly. They were preliminary but promising results, clearly a larger series of cases and prolonged follow-up are needed to clarify and confirm our observations.

## Introduction

Induced radiofrequency thermal ablation (RFA) is a minimally invasive technique developed to treat the symptoms and aesthetic outcomes of benign thyroid nodules. Thyroid nodules are the most common pathology of the thyroid gland, in most cases they are asymptomatic, stable over time and incidental; in a low percentage of cases, however, they have rapid growth associated with compression symptoms and unsightly outcomes (1). Until the last decade, these pathologies, when symptomatic, could only be treated with surgery. Recently, with the progress of minimally invasive techniques, thyroid nodules can be treated with various minimally invasive techniques including RFA (2).

RFA, performed by expert hands can be considered a safe and effective alternative to surgery for the treatment of selected pathologies. The RFA technique allows to obtain satisfactory results in terms of volumetric reduction of the nodule with a significant improvement in the quality of life. Patients treated with RFA ablation refer reduction of compression symptoms and discomfort, previously related to the presence of a mass in the neck. The trans-isthmic approach and the moving shot technique are the two basic techniques validated in many studies; however, an advanced technique, vascular radiofrequency ablation (vRFA), has been developed and validated (3).

Various International guidelines, indeed, consider RFA treatment a valid alternative to surgery in the case of symptomatic benign thyroid nodularities. Moreover, RFA may be an indication in the treatment of microcarcinoma, in recurrent thyroid cancers, in lymph node metastases and in cases where surgery is contraindicated or rejected by the patient (2–9). Especially in the treatment of papillary thyroid carcinoma recent data suggest the use of RFA as an alternative treatment to surgery (3, 4, 10–12).

The aim of this study is to evaluate the percentage of nodular volume reduction at 1 month, 3 months, and 6 months in patients treated with RFA and in patients treated with vRFA. The goal is to evaluate the difference between the two techniques in the time of reduction of the nodular volume and to identify the superiority of one technique compared to the other.

## Materials and Methods

We have prospectively collected data of 29 consecutive patients who underwent RFA or artery- first vRFA at Department of Endocrine and Ultrasound-guided Surgery of “Ospedale del Mare”, Naples, Italy. All patients had a diagnosis of nodular goiter and underwent radiofrequency ablation of thyroid nodule between January 2018 and August 2019.

**Figure 1:**
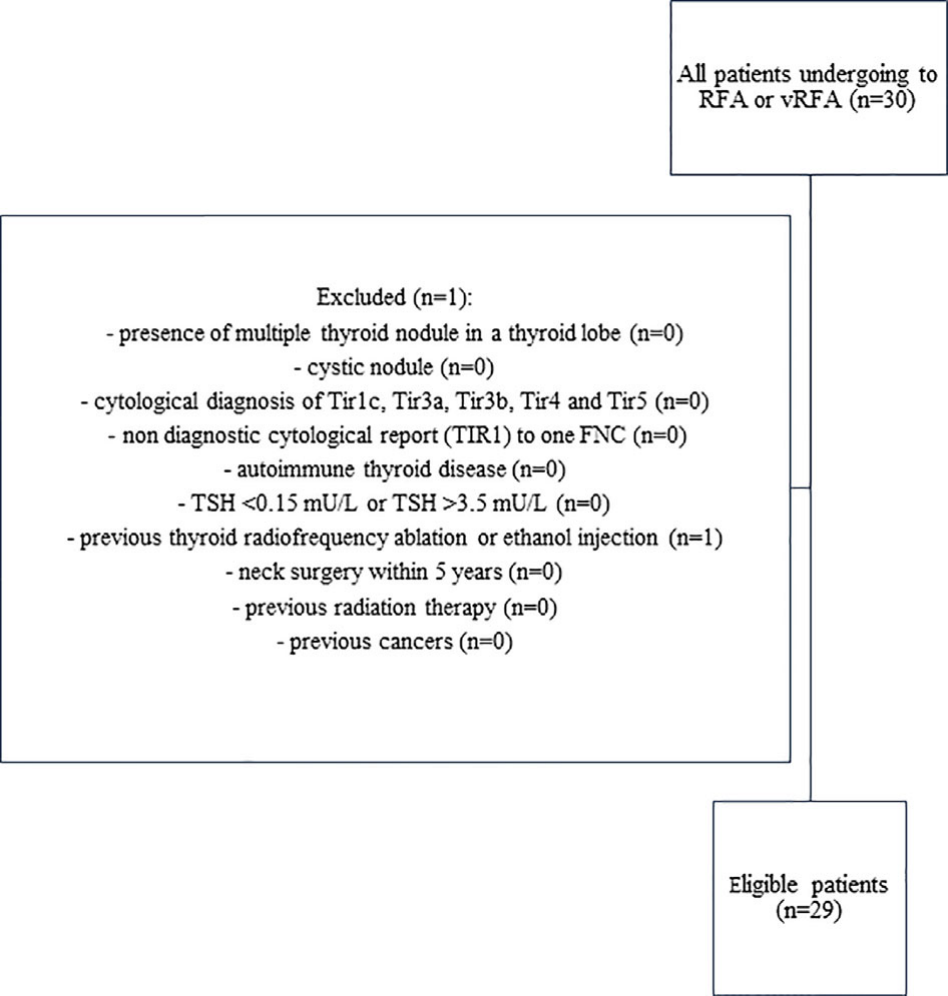
Flow chart of eligible patients.

We included patients aged over 18 years and not pregnant with: single thyroid nodule of the thyroid lobe with benign cytological diagnosis (Thy2/TIR2 or Bethesda II) to two consecutive fine needle cytology (FNC) (13, 14); complete medical history, complete preoperative blood tests and complete follow-up. We excluded patients with: presence of multiple thyroid nodule in a thyroid lobe; cystic nodules or predominantly cystic nodules on ultrasound (cystic portion >50% of the nodule); cytological diagnosis of TIR1c, TIR3a, TIR3b, TIR4 and TIR5; non diagnostic cytological report (TIR1) to one FNC; autoimmune thyroid disease; TSH <0.15 mU/L or TSH >3.5 mU/L; previous thyroid radiofrequency ablation or ethanol injection; neck surgery within 5 years; previous radiation therapy; previous cancers. The flow chart (**Figure 1**) shows the patients enrolled according to the inclusion and exclusion criteria.

We divided the population in two groups based on RFA technique used. Group A included RFA patients. Group B included, on the other hand, patients subjected to vRFA. All patients underwent coagulation index and blood cells count sample, thyroid function samples, thyroid antibody samples, twice FNC, thyroid and later cervical lymph nodes B-mode US examination and fiberoptic-laryngoscopy tests in the 30 days prior to ultrasonographic ablation techniques. Clinical and demographic data (age, gender, baseline nodule volume, nodule structure, nodule vascularization, nodule function and a complete anamnesis) were obtained at first examination and were collected in an electronic database.

The US examination was conducted with a 7.5–12 MHz linear probe or with a convex probe in very large nodules equipped with CD and PD nodules (MyLab™ClassC and MyLab™9 Platform, Esaote Biomedica, Genova Italy) and assessing the volume of the nodule, the structure and the vascularization. The basal volume of the nodule was automatically calculated with ellipsoid equation by the software with the measurements of depth, width and length in longitudinal and transverse scans. The structure was defined as solid (fluid component was less than 10% of volume), spongiform (cystic areas occupied less than 50% of the nodule’s volume) and predominantly solid (solid component was more than 50% of the volume). The vascularity was studied by CD examination and classified as peri-nodular, intra-nodular and both peri- and intra- nodular. Cytology findings, according to SIAPEC-IAP classification, were collected and we included patients with twice TIR2 at two consecutive FNCs.

The examined variables, relating to the technique, were the Joules (energy used to deliver the power of one watt for one second) used and the time expressed in seconds. Radiofrequency ablation was performed with the M-3004 generator and the RFT-TIP 0710N, 7 cm length, 1cm exposed tip (RF Medical Co., South Korea) inserted with US guidance.

The patients were submitted to a single RFA session, in supine position, in neck hyperextension and with a skin and pericapsular anesthesia. The skin local anesthesia was done in the needle insertion site in the midline. During the treatment, all patients had a feeling of compression at the neck’s level, which did not require the interruption of the procedure. All patients were discharged 3 h after the procedure, after ultrasound examination. No cases of major or minor complications were observed.

The trans-isthmic approach and the moving shot technique are performed under ultrasound (US) guidance. The trans-isthmic approach allows the treatment of the left and right thyroid nodules by inserting the RFA electrode in the neck midline to lateral direction. It shows advantages over other approaches. Before reaching the target, the electrode crosses much of the thyroid parenchyma limiting involuntary excursions of the needle-electrode due to patients’ involuntary movements (speaking, swallowing or coughing). It prevents the leakage of hot material from the thyroid parenchyma during the procedure. This approach also allows continuous ultrasound monitoring to minimize the risk of complications or side effects due to thermal injuries in surrounding structures (recurrent laryngeal nerve, carotid artery, internal jugular vein, trachea and esophagus) (5–7, 15–17). Moreover, the use of Color Doppler (CD) and/or Power Doppler (PD) prevents serious bleeding, identifying the blood vessels along the path of the electrode.

A radiofrequency generator (M-3004, RF Medical Co., Ltd., South Korea), connected to the needle-electrode, produces an alternating electric current (between 200 and 1,200 kHz) which causes ions movement in adjacent tissues of the tip. The ions vibrate rapidly producing energy that is transformed in heat, raising local temperature between 60°C and 100°C. The high temperature induces a protein denaturation of cell membrane and the irreversible death of the cells of the tissues around the tip. The energy decreases exponentially in the length of the needle and the remote tissues are heated due to thermal conduction. This needle protection mechanism prevents tissues away from the target from undergoing coagulation necrosis due to the heat generated (18). At the beginning of the procedure, the temperature necroses all around the tip. US transient hyperechoic area is displayed and to the touch a vibration is perceived, described as a “shot”.

The RFA has benefited from the moving shot technique as the thyroid nodules mainly have an ellipsoidal appearance and it is difficult to treat them with the fixed technique. The periphery of the thyroid nodules is undertreated or overtreated with the fixed technique. Using the moving shot technique, instead, the nodule is ideally divided into multiple smaller units, each to be ablated separately. The subunits are smaller on the periphery and larger in the central area and in regions far from critical structures. The procedure begins with the treatment of the deeper and more peripheral areas, as the heat generated causes the appearance of transient hyperechoic air bubbles that reduce the acoustic window for ultrasound monitoring, moreover, saving the “dangerous triangle” (the nodular periphery area close to trachea) to avoid thermal injury to the recurrent laryngeal nerve. It continues with the treatment of superficial areas of the nodule. The moving shot technique allows to obtain an ellipsoidal ablation area, similar to the morphology of the frequent thyroid nodules. The treatment ends when the entire nodule appears completely replaced by transient hyperechoic areas (3, 5, 10, 19–22).

The anesthetic infiltration of skin and pericapsular tissue is performed before the RFA: the needle is inserted at the midline of the anterior neck to anesthetize the skin. Once the skin has infiltrated, the needle is pushed in lateral direction, depending on the right or left position of the nodule, up to the virtual space between the strap muscles and the thyroid capsule. A solution with lidocaine 2% is injected and appears as an anechoic band, which gradually disappears deeply into antero-superior mediastinum, and laterally tools the main vessels of the neck. The risk of major complications decreases with local anesthesia, because the operator can monitor the pain, an early indicator of heat propagation, while a moderate sedation or a general anesthesia prevent the patients’ active collaboration and the early manifestation of complications such as changes in tone of voice, cough or pain (10, 15, 23).

The thyroid upper polar nodules are usually vascularized by arterial branches of the upper thyroid artery that derives from the external carotid artery and mainly supplies the upper and anterior part of the thyroid gland. The nodules of the lower pole of the thyroid are vascularized by the lower thyroid artery, which derives from the thyrocervical trunk and provides the postero-inferior parts of the gland.

**Figure 2:**
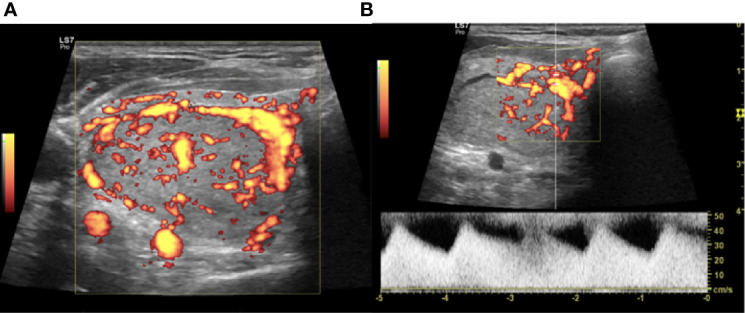
Right lobe nodule, intraoperative transverse ultrasound (US) scan. **(A)** Power Doppler (PD) evaluation of the nodule to be treated with vascular radiofrequency ablation (vRFA). **(B)** Identification at PD and PWD of the main afferent artery.

**Figure 3:**
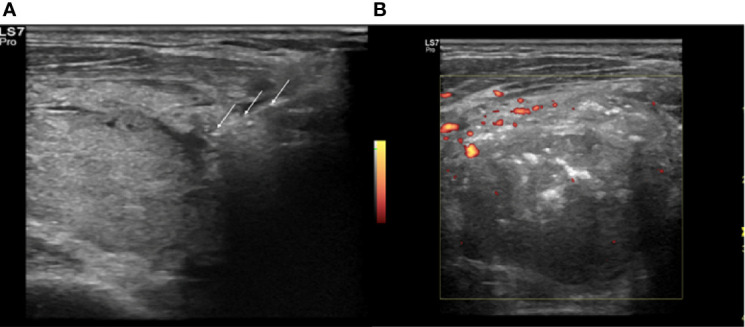
Right lobe nodule, intraoperative transverse ultrasound (US) scan. **(A)** Insertion of the electrode (white arrows) in the identified arterial afferent pole. **(B)** Power Doppler (PD) evaluation at the end of the procedure, absence of peri- and intranodular vasculartization.

The vRFA is a technique used to treat the hypervascular thyroid nodule. The artery-first vRFA is used in hypervascularized nodules that show an evident feeding artery. Authors propose this technique to reduce the dispersion of the generated heat, in the nodules with important vascularization (24). The feeding artery is detected with a CD and PD study (**Figure 2**) and the use of artery-first vRFA decreases the complications and increases the techniques efficiency. Intranodular linear echogenicities appear in the target nodule when the feeding artery is ablated due to air microbubbles in the arterioles (**Figure 3**). In a few cases, it’s possible to have wedge-shaped hypoechoic area after the ablation due to the infarction of the area vascularized by the ablated feeding artery (25).

**Figure 4:**
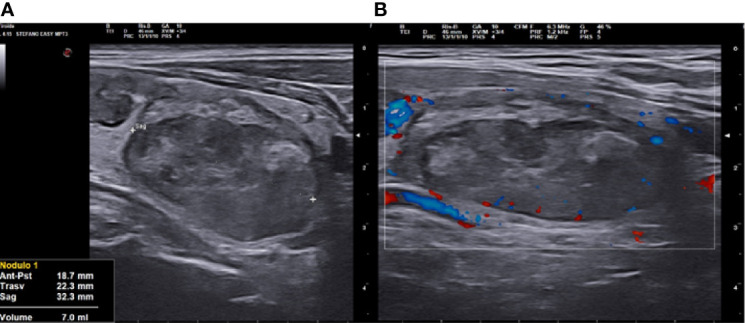
Right lobe nodule, longitudinal ultrasound (US) scan at 3 months follow up after vascular radiofrequency ablation (vRFA). **(A)** The nodule shrunk from 18.5 to 7 ml in 3 months, strongly hypoechoic, solid. **(B)** at Color Doppler (CD) evaluation, absence of peri- and intranodular vascular signal.

We carried out the thermo-ablative treatment of an afferent arterial branch of nodule as a first step of RFA treatment, improving the moving shot technique, in order to obtain a better ablation of nodular tissue, amplifying the volume of the induced necrosis (**Figure 4**) (3, 22, 25).

In the follow-up program we assessed the nodule volume and its percentage of reduction at 1 month, 3 months and 6 months. We assessed the volume with the ultrasound examination, evaluating the 3 diameters studied in the two projections. The reduction percentage was calculated comparing the follow-up volume with the basal volume.

Statistical analysis was performed with SPSS version 23 (SPSS^©^, Chicago, IL, USA). Continuous variables were described as mean, standard deviation (SD) and range, while categorical variables were described as number of cases and percentage. The population was divided in patients who underwent RFA and patients who underwent vRFA. Independent samples t-test were performed to compare the continuous variables (Joule, procedure’s time in second, basal volume, volume at 1 month, % of volume reduction at 1 month, volume at 3 months, % of volume reduction at 3 months, volume at 6 months and % of volume reduction at 6 months). A Test of Proportions was applied to the categorical variables (number of patients, nodule structure and vascularization). The Fisher’s exact test was used to analyze the gender. Statistical significance was considered in case of p-value <0.05.

Written informed consent was obtained from all participants. All procedures were performed in accordance with the Helsinki Declaration.

## Results

Twenty-nine patients (10 males and 19 females) with a single benign thyroid nodule were enrolled in the study for the treatment with radiofrequency ablation. Patients were divided into two groups based on the treatment they were subjected to, Group A treated with RFA (12 cases) and Group B treated with vRFA (17 cases). Population’s mean age was 55.03 years (± 10.9 SD) without a statistical difference between two groups. Patients included were euthyroid and they had normal values of Thyroglobulin Antibody (TgAb), Thyroperossidase Antibody (TPOAb) and calcitonin (**Table 1**). In a case of subclinical hyperthyroidism (TSH 0.12 mU/L) thyroid scintigraphy was performed prior to treatment and it identified a non-functioning nodule. The mean Thyroid-stimulating hormone (TSH) value was 1.03 mU/L (± 0.49 mU/L SD) and showed no significant differences between the two populations (**Table 1**).

**Table 1:** Baseline demographic and clinical data of the study population.

*Characteristics*	Population	Group A	Group B	p-value
***Patients (%)***	29 (100)	12 (41.4)	17 (58.6)	0.14
***Mean age in years ± SD (range)***	55.03 ± 10.90 (28–80)	57.50 ± 7.28 (46–69)	53.29 ± 12.80 (28–80)	0.315
***Gender (%)***				
***Male*** ***Female***	10 (34.5) 19 (65.5)	4 (13.8) 8 (86.2)	6 (20.7) 11 (79.3)	0.99
***Mean TSH in mU/L ± SD (range)***	1.03 ± 0.49 (0.12–2.21)	0.93 ± 0.42 (0.54–1.78)	1.11 ± 0.54 (0.12–2.21)	0.337
***Mean basal volumes ± SD (range)***	26.90 ± 19.09 (1.60–74)	21.15 ± 20.34 (1.60–74)	30.96 ± 17.65 (10.40–70.10)	0.178
***Nodule structure (%)***				
***Solid*** ***Spongiform*** ***Predominantly solid***	9 (31) 18 (62) 2 (7)	7 (58) 4 (33.5) 1 (8.5)	2 (11.8) 14 (82.4) 1 (5.8)	0.023 0.022 0.99
***Nodule vascularization (%)***				
***Peri-nodular*** ***Intra-nodular*** ***Intra-peri-nodular***	8 (27.6) 1 (3.5) 20 (68.9)	5 (41.7) 1 (8.5) 6 (49.8)	3 (17.7) 0 (0) 14 (82.3)	0.315 0.858 0.14

SD, standard deviation; TSH, thyro-stimulating hormone.

Ultrasound nodule structure was summarized in **Table 1**. Nodules with solid, spongiform and predominantly solid structure were selected. Solid structure and spongiform structure showed statistically significant differences with p-values of 0.022 and 0.023 respectively. The solid structure nodules were treated more with RFA (seven cases with RFA versus two cases with vRFA), while the spongiform nodules were treated more with vRFA (four cases with RFA versus 14 cases with vRFA).

Baseline nodule vascularization characteristics are summarized in **Table 1** and do not show significant difference.

At baseline, the nodule volume ranged 1.60 to 74 ml with a mean of 26.90 ± 19.09 ml (**Table 1**). Considering the two groups separately, the Group A’ mean volume was 21.15 ± 20.34 ml, in the other hand, Group B’ mean volume was 30.96 ± 17.65 ml with a p-value=0.178. The variables of the ablative techniques are illustrated in **Table 2**. The Joules/energy used showed a statistically significant difference between the two techniques, with a mean of 29663.83 ± 21040.14 J/s in Group A and a mean of 48275.47 ± 26029.28 J/s (p-value=0.05), showing that more energy must be used in vRFA. The execution time expressed in seconds showed no significant difference. Both techniques were conducted by two skilled operators of a tertiary thyroid center.

**Table 2:** Radiofrequency ablation characteristics (SD, standard deviation).

*Characteristics*	Population	Group A	Group B	p-value
***Mean used Joule ± SD (range)***	40,574.10 ± 25,457.69 (4,560–98,000)	29,663.83 ± 21,040.14 (4,560–71,180)	48,275.47 ± 26,029.28 (9,800–98,000)	0.05
***Mean time in seconds ± SD (range)***	638.55 ± 299.33 (192–1,320)	537.58 ± 244.47 (192–985)	709.82 ± 320.42 (204–1,320)	0.129

**Figure 5:**
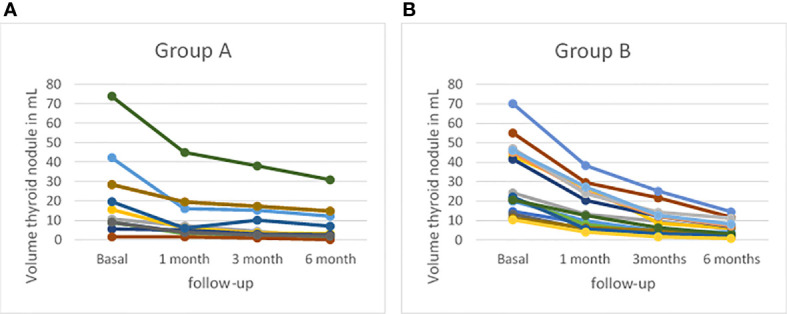
Profiles of thyroid volume assessed by B-mode ultrasound (US) at baseline and at 1-, 3-, and 6-month follow-up **(A)** in patients treated with radiofrequency thermal ablation (RFA) (Group A) and **(B)** in patients treated with vRFA (Group B).

After radiofrequency ablation treatment the mean volume of nodule decreased progressively (**Table 3**, **Figure 5**): it was 14.15 ± 11.37 ml 1 month after treatment; 9.26 ± 8.48 ml 3 months after treatment and 6.41 ± 6.53 ml 6 months after treatment (p-value of 0.279, 0.784 and 0.451, respectively). The nodule volume decreased gradually during 1 month of follow-up with a mean of 14.15 ml versus 26.9 ml at baseline. The percentage of reduction at 1 month was 47.26% in study population without a significant difference between Group A and Group B (p-value= 0.369). Subsequently, percentage of volume reduction was decreased mostly in the Group B at 3 months and 6 months of follow-up with a p-value of 0.003 and 0.013, respectively. At 3 months of follow-up, Group B showed a mean percentage reduction of 72.71 ± 8.99% compared to the Group A’s mean percentage reduction of 56.28 ± 14.55%. Moreover, at 6 months follow-up, Group B showed a mean percentage reduction of 83.01 ± 5.04% compared to Group A’s mean percentage reduction of 70.04 ± 15.04%. Data showed that the percentage of nodule reduction was not dissimilar between the two groups after one month from the ablative treatment. At 3 months, instead, there was a significant percentage of major reduction in patients treated with vRFA. This statistical significance remained unchanged at 6 months, registering a greater volume reduction percentage in patients treated with vRFA than in patients treated with RFA.

**Table 3:** Thyroid nodule volume in follow-up (SD, standard deviation).

*Characteristics*	*Population*	*Group A*	*Group B*	*p-value*
***1 month volume ± SD (range)***	14.15 ± 11.37 (1.60−45)	11.38 ± 12.36 (1.60−45)	16.09 ± 10.55 (4.12−38.40)	0.279
***1 month % volume variation ± SD (range)***	47.26 ± 16.5(0−74.26)	43.45 ± 22.67 (0−69.54)	49.95 ± 10.23 (35.58−74.26)	0.369
***3 months volume ± SD (range)***	9.26 ± 8.48 (0.95−38)	9.78 ± 10.8 (0.95−38)	8.88 ± 6.73 (1.60−25)	0.784
***3 months % volume variation ± SD (range)***	65.91 ± 14.04 (36.64−85.88)	56.28 ± 14.55 (36.64−76.93)	72.71 ± 8.99 (57.14−85.88)	0.003
***6 months volume*** *±* ***SD (range)***	6.41 ± 6.53 (0.21−31)	7.68 ± 9.11 (0.21−31)	5.51 ± 3.92 (0.90−14.40)	0.451
***6 months % volume variation*** *±* ***SD (range)***	77.64 ± 12.07 (48.07−91.34)	70.04 ± 15.04 (48.07−89.72)	83.01 ± 5.04 (75.89−91.34)	0.013

## Discussion

Numerous studies have shown that radiofrequency ablative treatment is an effective and safe treatment in symptomatic benign nodules of the thyroid (1, 12, 16, 19, 21, 26). International guidelines show that radiofrequency is effective in the treatment of microcarcinomas and recurrent carcinomas of less than 2 cm in diameter, not only in the treatment of symptomatic benign nodules (2, 10). Any study has shown the difference in the efficacy of RFA versus vRFA with artery-first step ablation techniques. The aim of our study was to evaluate the difference in the effectiveness of RFA and vRFA with a 6-month follow-up in symptomatic benign thyroid nodule.

Effective cytoreduction occurs when large volumes of necrosis are rapidly obtained with ablative treatments (22). In our population study, the percentage of volume reduction was similar between the two groups at 1-month follow-up, but it’s statistically different in Group B at 3- and 6-months follow-up. The percentage of reduction at 1-month is not different in the two groups due to infarction of the nodule treated with vRFA. The infarction is due to a reduction in the vascularization of the nodule and to a reduction in venous drainage (3). This scenario changes at 3 and 6 months of follow-up, in which there is a reduction in inflammation due to necrosis and a resumption of venous drainage with the removal of the necrotic cells from the treated nodule. Some Authors suggest that a volumetric reduction of treated nodule is from 50 to 80% after 6 months and from 70 to 90% after two years. In our cases we had a volume reduction of 43.45%, 56.28%, and 70.04% at 1-, 3-, and 6- months in Group A and a volume reduction of 49.95%, 72.71%, and 83.01% at 1-, 3-, and 6-months in Group B. The percentage of volume reduction is in line with the Literature data with a reduction of more than 50% of the volume already after 3 months of treatment in both techniques (21). In the future we will publish a study with a wider case history and a longer follow-up, to evaluate the percentage of reduction over the years and a possible regrowth of the nodule, comparing the two methods. The long-term efficacy of the treatment is assessed by observing the absence of nodular vital tissue regrowth, which can be demonstrated during ultrasound examinations carried out in the period following the treatment. Nodular regrowth, in the other hand, has been associated with the presence of inadequately treated nodular margins (21, 26–28).

In our population study, the nodules’ feeding artery is ablated with RFA before starting the ablative treatment. The rational of RFA is to increase the local temperature in order to induce tissue necrosis. Many benign thyroid nodules are hyper-vascularized. The adjacent vascular structures can dissipate the heat generated during the procedure reducing the treatment’s effectiveness. The early ablation of afferent artery reduces the vascularization of the nodule and the heat dissipation. Moreover, the afferent artery ablation reduces the risk of bleeding and of hematomas. The hemorrhage, during the RFA, leads to increase of volume and to obstacle the thermal conduction (3, 22). Our population shows a difference compared to the structure of the nodule. In Group A we have 58% of the population with a solid echostructure of the nodule, while in the Group B we have 82.4% with a spongiform echostructure of the nodule. The significant difference is affected by the choice of technique. vRFA can be used in the treatment of nodules with a feeding artery visible and nodules with a spongiform echostructure are nodules of greater size and intensely vascularized. Deandrea et al. suggest that spongiform nodule and cystic nodule have a greater reduction volume compared to solid nodules (21). Deandrea et al. hypothesize that in the nodules with a greater fluid component and in the more vascularized ones, the heat induced by the electrode produces more steam and the steam improves the thermocoagulation process (21). These data are in line with the ones observed in our study population. The energy utilized (J/s) shows significant difference. In vRFA group we have utilized increased energy (29663.83 vs 48275.47 J/s) as the method foresees two treatment steps: a first phase in which the nutritious artery is ablated and a second phase in which the nodule is ablated.

Park et al. describe an US hyperechoic linear streak at the nodule periphery after the afferent artery ablation. The US image is due to overheating which induces the appearance of microbubbles inside the arterioles. In some cases, on the other hand, it’s possible to view a wedge-shaped hypoechoic area due to ischemia. The ischemic area is a rare finding especially in hypervascularized nodules due to the presence of several collateral circles (3).

Our preliminary experience, conducted on symptomatic thyroid nodules, shows that the radiofrequency treatment is a safe and an effective treatment. The method allows to reduce the compression symptoms, to obtain high percentages of reduction of the volume of the nodule even with just one treatment, to treat the nodule with the most effective technique based on the anatomical conditions and is squeezed to a risk of complications absent when performed by expert hands.

We achieved a significant percentage of greater volume reduction in the vRFA group compared to patients treated only with RFA. At follow-up, peripheral vascularization appears less evident on CD, in patients undergoing vRFA than in those treated only with RFA. These considerations allow us to hypothesize that vRFA may improve the effectiveness of the procedure, allowing for an improvement in symptoms and a reduction in volume more quickly. They were preliminary but promising results, clearly a larger series of cases and prolonged follow-up are needed to clarify and confirm our observations.

## Data Availability Statement

The raw data supporting the conclusions of this article will be made available by the authors, without undue reservation.

## Ethics Statement

The studies involving human participants were reviewed and approved by Ethics Committee Campania Center Prot. no 375/C.E 18-2020 oss of 09/15/2020. The patients/participants provided their written informed consent to participate in this study.

## Author Contributions

All authors contributed significantly to the present research and reviewed the entire manuscript. CO participated substantially in conception, design and execution of the study and in the analysis and interpretation of the data; also participated substantially in the drafting and editing of the manuscript. SG participated substantially in conception, design and execution of the study and in the analysis and interpretation of the data; also participated substantially in the drafting and editing of the manuscript. GA participated substantially in conception and design of the manuscript and in the analysis and interpretation of the data. ME participated substantially in conception and design of the manuscript and in the analysis and interpretation of the data. UB participated substantially in conception and design of the manuscript and in the analysis and interpretation of the data. CM participated substantially in conception and design of the manuscript and in the analysis and interpretation of the data. ED’A participated substantially in conception and design of the manuscript and in the analysis and interpretation of the data. DP participated substantially in conception and design of the manuscript and in the analysis and interpretation of the data. SS participated substantially in conception, design and execution of the study and in the analysis and interpretation of the data; also participated substantially in the drafting and editing of the manuscript. All authors contributed to the article and approved the submitted version.

## Conflict of Interest

The authors declare that the research was conducted in the absence of any commercial or financial relationships that could be construed as a potential conflict of interest.

## Abbreviations

CD, Color Doppler; FNC, fine needle cytology; PD, Power Doppler; RFA, radiofrequency ablation; SD, standard deviation; TgAb, thyroglobulin antibody; TPOAb, Thyroperossidase antibody; TSH, thyro-stimulating hormone; US, ultrasound; vRFA, vascular radiofrequency ablation.
